# *sarA*-Dependent Antibiofilm Activity of Thymol Enhances the Antibacterial Efficacy of Rifampicin Against *Staphylococcus aureus*

**DOI:** 10.3389/fmicb.2020.01744

**Published:** 2020-07-31

**Authors:** Alaguvel Valliammai, Anthonymuthu Selvaraj, Udayakumar Yuvashree, Chairmandurai Aravindraja, Shunmugiah Karutha Pandian

**Affiliations:** ^1^Department of Biotechnology, Alagappa University, Karaikudi, India; ^2^Department of Periodontology, College of Dentistry, University of Florida, Gainesville, FL, United States

**Keywords:** MRSA, thymol, rifampicin, *sarA*, biofilm inhibition, biofilm eradication

## Abstract

Methicillin-resistant *Staphylococcus aureus* (MRSA) is a serious human pathogen which has been listed as a high-priority multi-drug resistance pathogen by the World Health Organization (WHO). Persistent MRSA infections are often associated with biofilm formation and resistance to conventional antimicrobial therapy. Inhibiting the surface adherence and the virulence of the bacterium is the current alternative approach without affecting growth to reduce the possibility of resistance development. Although numerous antibiofilm agents have been identified, their mode of action remains unclear. Combining two drugs with different modes of action will improve the efficiency of the treatment strategy against MRSA. The present study was aimed to decipher the molecular mechanism underlying the antibiofilm activity of thymol against MRSA and assess the ability of thymol to improve the antibacterial activity of rifampicin. Thymol significantly inhibited 88% of MRSA biofilm formation at 100 μg/ml and reduced the surface adherence of MRSA on glass, stainless steel, and titanium surface coated with human plasma as evidenced by microscopic analyses. qPCR analysis of global virulence regulatory genes and biofilm assay with *S. aureus* wild type, Δ*sarA*, and Δ*agr* strains revealed the *sarA*-mediated antibiofilm activity of thymol and inhibition of *sarA*-controlled virulence factors. Congo red assay and erythrocyte lysis assay further confirmed the reduction in polysaccharide intracellular adhesin and hemolysin. Importantly, thymol enhanced the antibacterial and the biofilm eradication efficiency of rifampicin against MRSA and also reduced the formation of persisters. Thus, the present study reveals the *sarA*-dependent antibiofilm efficacy of MRSA and suggests thymol as the promising combinatorial candidate in potentiating the antibacterial activity of rifampicin against persistent MRSA infections.

## Introduction

*Staphylococcus aureus*, a Gram-positive human commensal bacterium of the nasal epithelium turns virulent when the individual is immune-compromised. *S. aureus* not alone causes simple skin infections but also causes lethal infections such as pulmonary infections, invasive endocarditis, septic arthritis, osteomyelitis, and peri-implantitis (Taylor and Unakal, 2020). The treatment of these lethal conditions is a challenging task due to evolving multi-drug-resistant strains such as methicillin-resistant *S. aureus* (MRSA). MRSA is majorly involved in community-associated, hospital-associated as well as livestock-associated infections (Palavecino, 2014). Numerous virulence traits of MRSA make it stubborn against the conventional antibiotic therapy. Biofilm formation is one such trait which enables the bacterium to survive against various physiological stresses. Bacterial cells adhered to a surface with the help of slimy polymeric substances called biofilm, and this biofilm provides multicellular behavior to the unicellular bacterial cells. The rate of gene transfer inside the biofilm is higher than that between planktonic cells (Kumar et al., 2019). Bacterial cells especially living in biofilm mode are having a peculiar behavior of synchronized virulence gene expression, and many enzymes present in biofilm matrix are reported to cleave the antibiotics. In addition, the biofilm blocks the penetration of antibiotics as it is slimy and mucus in nature. Altogether the bacterial cells residing in biofilm are much more resistant to antibiotics than their planktonic counterparts (Olsen, 2015; Hall and Mah, 2017).

Bacterium has an intricate regulatory network to coordinate the synthesis of virulence factors. Although various global regulatory systems have been identified in *S. aureus*, the accessory gene regulatory (*agr*) system and the staphylococcal accessory regulatory system (*sarA*) are prototypes in nature. More specifically, both systems are well known to regulate biofilm formation and virulence factor production in a reciprocal way (Jenul and Horswill, 2018). That is, the active *agr* system negatively regulates the adhesion genes responsible for biofilm formation and leads to biofilm dispersal, whereas the active *sarA* system enhances the biofilm formation. Hence, *agr* and *sarA* systems act as molecular switches in regulating biofilm formation in *S. aureus* (Vasudevan, 2019).

As biofilm plays a critical role in the development of antibiotic resistance, targeting biofilm formation has become an alternative strategy to antibiotics (Buommino et al., 2014). Notably, antibiofilm agents are reported to potentiate the efficacy of antibiotics against bacterial biofilm when combined with antibiotics. Plenty of compounds with antibiofilm activity have been identified from various natural resources so far (Abraham et al., 2012; Sethupathy et al., 2016, 2017; Selvaraj et al., 2019; Valliammai et al., 2019). Thymol is a major constituent in the essential oil of thyme plant (*Thymus vulgaris*), and it is known to have various biological properties such as antibacterial, antifungal, antioxidant, and cognitive-enhancing activities (Braga, 2005; Tohidpour et al., 2010; Azizi et al., 2012). Few reports are available on the antibiofilm activity of thymol against *S. aureus*, but the molecular mechanism underlying the biofilm inhibitory potential of thymol remains unclear (Nostro et al., 2007; García-Salinas et al., 2018). Henceforth, the goal of the present study is to unravel the molecular mechanism of the antibiofilm efficacy of thymol and to find out the ability of thymol to improve the efficacy of rifampicin.

## Materials and Methods

### Bacterial Strains and Growth Conditions

The *S. aureus* strains used in the present study are listed in Table 1. The reference MRSA strain used throughout the study was obtained from the American Type Culture Collection (ATCC). The clinical isolates were collected from pharingitis patients at the Rajaji Government Hospital, Madurai, Tamil Nadu for our earlier work (Gowrishankar et al., 2012). Newman wild-type and mutant strains of *S. aureus* were gifted by Dr. Christiane Wolz, a professor at the Institute for Medical Microbiology and Hygiene, University of Tubingen, Germany. For the biofilm assays, all the bacterial strains were cultured in tryptone soya broth (TSB) supplemented with 1% sucrose (TSBS) and kept at 37°C in the shaking incubator for 24 h. For maintenance, the bacterial cultures were grown in TSB and stored as glycerol stocks at −80°C.

**TABLE 1:** *Staphylococcus aureus* strains used in the present study.

**Strain name**	**Details**
MRSA	ATCC 33591
MSSA 46	Clinical isolate (Genebank ID: JN315153)
MSSA 51	Clinical isolate (Genebank ID: JN315154)
MRSA 44	Clinical isolate (Genebank ID: JN315148)
*S. aureus*	Newman wild-type strain
Δ*sarA*	ALC 637-Newman Δ*sarA*:*Tn917LTV1*
Δ*agr*	ALC 355-Newman Δ*agr*:*tetM*

### Stock Solution

Thymol was purchased from Sigma-Aldrich, India. Ten milligrams of thymol dissolved in 1 ml of methanol was used as stock solution (10 mg/ml). Methanol alone was used as vehicle control in all the assays.

### Determination of Biofilm Inhibitory Concentration

A 24-well polystyrene microtiter plate (MTP) containing 1 ml of TSBS with various concentrations of thymol (20–200 μg/ml) was inoculated with 1% of 6-h cultures of *S. aureus* strains (1 × 10^8^ cells) and incubated at 37°C for 24 h. Methanol was used as vehicle control. For biofilm quantification, planktonic cells were carefully removed and biofilm cells were washed with sterile saline solution to remove unbound cells, and the plate was air-dried. Then, the biofilm cells were stained with 0.4% crystal violet solution for 20 min, washed to remove the excess stain, and air-dried. For quantification, the biofilm cells were destained with 30% glacial acetic acid solution, and absorbance was read at 570 nm using a multi-label reader (Spectramax M3, United States). The percentage of biofilm inhibition was calculated using the formula:

%ofinhibition=[(controlOD-570⁢nmtreatedOD)570⁢nm /controlOD]570⁢nm×100.

The lowest concentration of thymol which exhibited maximum biofilm inhibition was set as the biofilm inhibitory concentration (BIC) (Valliammai et al., 2019).

### Colony-Forming Unit and Alamar Blue Assay

Two milliliters of MRSA cultures grown in the absence and the presence of thymol (25, 50, and 100 μg/ml) for 24 h was measured for absorbance at 600 nm. The control and the treated cultures were serially diluted and spread on a plate for colony-forming unit (CFU) counting. Then, the control and the treated cells were pelletized by centrifugation and dispersed in 2 ml of phosphate-buffered saline (PBS). A total of 900 μl of this suspension was aliquoted into wells of a 24-well MTP and 100 μl of Alamar blue solution (6.5 mg/ml) was added to each well. This plate was kept in the dark for 4 h, and fluorescence was measured at 530 and 590 nm for excitation and emission, respectively (Sarker et al., 2007).

### Light and Confocal Laser Scanning Microscopic Analyses

For microscopic analysis, biofilm was allowed to form on glass/stainless steel slides (1 cm × 1 cm) immersed in TSBS in the absence and the presence of thymol (25, 50, and 100 μg/ml) for 24 h at 37°C. After 24 h, the glass slides were taken out and washed with sterile saline solution. For the light microscopic analysis, the glass slides were stained with 0.4% crystal violet for 20 min and washed. After drying, the glass slides were observed under a light microscope (Nikon Eclipse 80i, United States) at ×400 magnification. For confocal laser scanning microscopic analysis, stainless steel slides were stained with 0.1% acridine orange in the dark for 20 min and washed. After drying, the stainless steel slides were observed under CLSM (LSM 710, Carl Zeiss, Germany) at ×200 magnification (Valliammai et al., 2019).

### Plasma Coating on Titanium Surface and Scanning Electron Microscopy Analysis

Plasma extracted from healthy human blood was diluted to 20% concentration using 50 mM sodium bicarbonate solution. Titanium slides (1 cm × 1 cm) placed in a 24-well MTP were covered with 1 ml of the prepared plasma solution and incubated at 4°C overnight. After the incubation period, the plasma solution was removed and the titanium slides were washed with sterile distilled water. Then, biofilm was allowed to form on the titanium surface in the absence and the presence of thymol (25, 50, and 100 μg/ml) as mentioned earlier. After 24 h of biofilm formation, the titanium slides were taken out and washed. For SEM analysis, the titanium slides were fixed with 2% glutaraldehyde at 4°C overnight and dehydrated with increasing concentrations of ethanol (20, 40, 60, 80, and 100%). After drying, the titanium slides were subjected to gold sputtering and observed under SEM (VEGA 3 TESCAN, Czech Republic) (Walker and Horswill, 2012).

### qPCR Analysis

Total RNA from 24-h control and thymol (100 μg/ml)-treated MRSA cultures was extracted using the TRIzol method of RNA extraction and converted to cDNA using High Capacity cDNA Reverse Transcription Kit (Applied Biosystems, United States). The quantification of gene expression in the control and the thymol-treated samples was performed in triplicate on a thermal cycler (7500 Sequence Detection System, Applied Biosystems Inc., Foster, CA, United States) using a PCR mix (SYBR Green Kit, Applied Biosystems, United States) at a predefined ratio. The fold change in gene expression was calculated by the 2^–ΔΔCt^ method with gyrB as the housekeeping gene (Livak and Schmittgen, 2001). The details of the primer sequences of the genes (*agrA*, *agrC*, *sarA*, *icaA*, *icaD*, *fnbA*, *fnbB*, and *hla*) used in this study are given in Table 2.

**TABLE 2:** List of primers used for qPCR analysis.

**Genes**	**Forward primer**	**Reverse primer**
*agrA*	5′-TGATAATCCTTATGAGGTGCTT-3′	5′-CACTGTGACTCGTAACGAAAA-3′
*agrC*	5′-CATTCGCGTTGCATTTATTG-3′	5′-CCTAAACCACGACCTTCACC-3′
*sarA*	5′-CAAACAACCACAAGTTGTTAAAGC-3′	5′-TGTTTGCTTCAGTGATTCGTTT-3′
*fnbA*	5′-ATCAGCAGATGTAGCGGAAG-3′	5′-TTTAGTACCGCTCGTTGTCC-3′
*fnbB*	5′-AAGAAGCACCGAAAACTGTG-3′	5′-TCTCTGCAACTGCTGTAACG-3′
*icaA*	5′-ACACTTGCTGGCGCAGTCAA-3′	5′-TCTGGAACCAACATCCAACA-3′
*icaD*	5′-ATGGTCAAGCCCAGACAGAG-3′	5′-AGTATTTTCAATGTTTAAAGCA-3′
*hla*	5′-CAACTGATAAAAAAGTAGGCTGGAAAGTGAT-3′	5′-CTGGTGAAAACCCTGAAGATAATAGAG-3′
*gyrB*	5′-GGTGCTGGGCAAATACAAGT-3′	5′-TCCCACACTAAATGGTGCAA-3′

### Biofilm Assay With Mutant Strains

As stated earlier, a 24-well MTP assay was performed with the *S. aureus* wild-type strain and isogenic Δ*sarA* and Δ*agr* strains. All these strains were treated with increasing concentrations of thymol (20–100 μg/ml) for 24 h at 37°C and observed for biofilm inhibition using crystal violet as mentioned.

### Congo Red Agar Assay

Tryptone soya broth supplemented with 1% sucrose containing 2% bacteriological agar and 0.08% Congo red was prepared and sterilized. After sterilization, thymol (25, 50, and 100 μg/ml) or methanol was mixed with the media and poured into Petri plates. The MRSA culture was streaked on the agar plates with and without thymol and incubated at 37°C for 24 h. After 24 h of incubation, the plates were photographed (Knobloch et al., 2002).

### Extracellular Polysaccharide Quantification

Biofilm formation assay was done in the absence and the presence of thymol (25, 50, and 100 μg/ml) as stated earlier. After 24 h of biofilm formation, planktonic cells were discarded and the biofilm matrix was scraped and collected using sterile PBS. To the collected biofilm, an equal volume of 5% phenol and five volumes of concentrated sulfuric acid containing 0.2% hydrazine sulfate were added and mixed well. This mixture was incubated in the dark at room temperature for 1 h. Then, the samples were centrifuged at 10,000 rpm for 10 min to collect the supernatants, and absorbance was measured at 490 nm (Dubois et al., 1951).

### Erythrocyte Lysis Assay

The 48-h MRSA cultures grown in the absence and the presence of thymol (25, 50, and 100 μg/ml) were centrifuged to collect cell-free culture supernatant (CFCS). Then, 100 μl of CFCS was mixed with 900 μl of 2% human red blood cell suspension prepared in PBS. This mixture was incubated at 37°C for 1 h and centrifuged at 10,000 rpm for 10 min. The collected supernatant was read for absorbance at 540 nm (Bernheimer, 1988).

### Determination of Minimum Inhibitory Concentration and Combinatorial Growth Inhibition Assay

Test tubes containing 2 ml of TSB with 1% MRSA cells (1 × 10^8^ cells) were treated with either thymol (0–200 μg/ml) or rifampicin (0–1 μg/ml) for 24 h at 37°C. After incubation, growth optical density (OD) at 600 nm was measured to determine the BIC. For the combinatorial assay, MRSA cells were treated with rifampicin (0.015, 0.03, and 0.06 μg/ml) in the presence and the absence of thymol (100 μg/ml) and incubated at 37°C for 24 h. After incubation along with growth OD at 600 nm, metabolic viability was also measured using Alamar blue assay as mentioned previously (Sarker et al., 2007).

### Disc Diffusion Assay

Methicillin-resistant *Staphylococcus aureus* cells (5 × 10^8^ cells) were spread on the surface of tryptic soya agar (TSA) plates without and with thymol (100 μg/ml) using sterile cotton swabs. The sterile discs were placed in the center of the agar surface and loaded with increasing concentrations of rifampicin (0.015, 0.03, and 0.06 μg/ml). The agar plates were incubated at 37°C for 24 h and photographed.

### Time Killing Assay

Methicillin-resistant *Staphylococcus aureus* cultures (1 × 10^8^ cells) were treated with rifampicin (0.06 μg/ml) and/or thymol (100 μg/ml), and 5 μl of the control and the treated cultures was spotted on the TSA plates every 1 h. In addition, 100 μl of the control and the treated cultures was spread on TSA plates every 2 h after serial dilution for CFU counting. The agar plates were incubated for 24 h at 37°C and photographed (Poonacha et al., 2017).

### Persister Formation Assay

An overnight culture of MRSA was diluted to 1:1,000 ratio using TSB and incubated at 37°C in the shaking incubator at 250 rpm to reach an exponential phase (0.8 OD at 600 nm). Then, the exponential-phase MRSA cultures were challenged with ×100 concentration of rifampicin (6 μg/ml) in the absence and the presence of thymol (25, 50, and 100 μg/ml) and incubated at 37°C in the shaking incubator at 250 rpm for 3 h. After incubation, 100 μl of the culture was spread on TSA, and persister cell survival was determined by counting the number of colonies formed on TSA after incubation for 24 h at 37°C (Lee et al., 2016).

### Biofilm Eradication Assay

Methicillin-resistant *Staphylococcus aureus* cells were allowed to form a biofilm in a 24-well MTP for 24 h without any treatment. The preformed biofilm was then treated with rifampicin (0.06 μg/ml) and/or thymol (100 μg/ml) for 12 h. After treatment, the planktonic cells were discarded and the biofilm cells were scraped out using sterile PBS and assessed for viability using Alamar blue as mentioned earlier. Additionally, the biofilm cells were serially diluted and plated for CFU counting.

### Statistics

All the experiments were carried out in three biological replicates with at least two experimental replicates, and the results were expressed as mean ± standard deviation. Statistical analysis was performed using SPSS 17.0 software package (SPSS Inc., Chicago, IL, United States), and one-way ANOVA followed by Duncan’s *post hoc* test was used to assess the significance. A *p*-value ≤ 0.05 was set as statistically significant.

## Results

### Thymol Inhibits MRSA Biofilm Formation Without Affecting Growth

**FIGURE 1:**
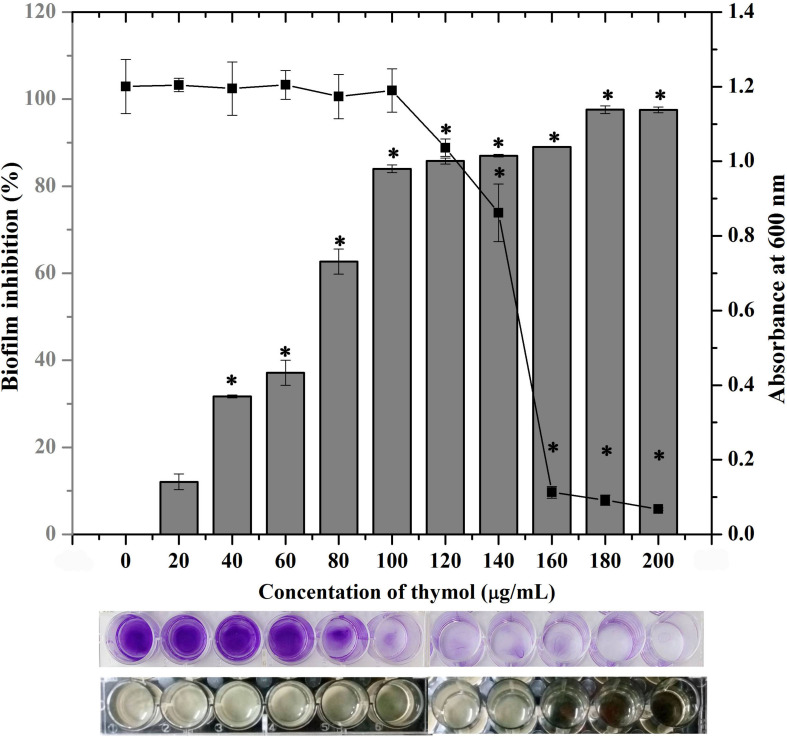
Effect of thymol on the biofilm formation of methicillin-resistant *Staphylococcus aureus* (MRSA) as assessed by crystal violet staining. The bar graph indicates the percentage of biofilm inhibition and the line graph indicates the growth at OD_600 nm_. The bottom images show the corresponding crystal violet-stained biofilm and the growth of MRSA in microtitre plate. The error bars indicate standard deviations. The asterisks represent statistical significance (*p* < 0.05).

The crystal violet quantification of biofilm formed in the absence and the presence of various concentrations of thymol (20–200 μg/ml) exhibited a dose-dependent antibiofilm activity with maximum biofilm inhibition of 88% at 100 μg/ml without affecting growth. Beyond this concentration, thymol started to affect the growth of MRSA and biofilm inhibition was not much increased. Hence, 100 μg/ml of thymol was considered as the BIC and taken for further assays (Figure 1). The same concentration of thymol was also found to inhibit the biofilm formation of clinical isolates, namely, MSSA-51, MSSA-46, and MRSA-44 (Supplementary Figure S1). CFU analysis and growth OD demonstrated the non-antibacterial activity of thymol at 25, 50, and 100 μg/ml (Supplementary Figure S2A). In the Alamar blue assay, the metabolic viability of thymol-treated MRSA cells was comparable to that of the control cells, confirming that thymol did not affect the metabolic viability of MRSA (Supplementary Figure S2B).

### Thymol Impedes the Adherence of MRSA on Glass, Stainless Steel Slides, and Titanium Slide Coated With Human Plasma

**FIGURE 2:**
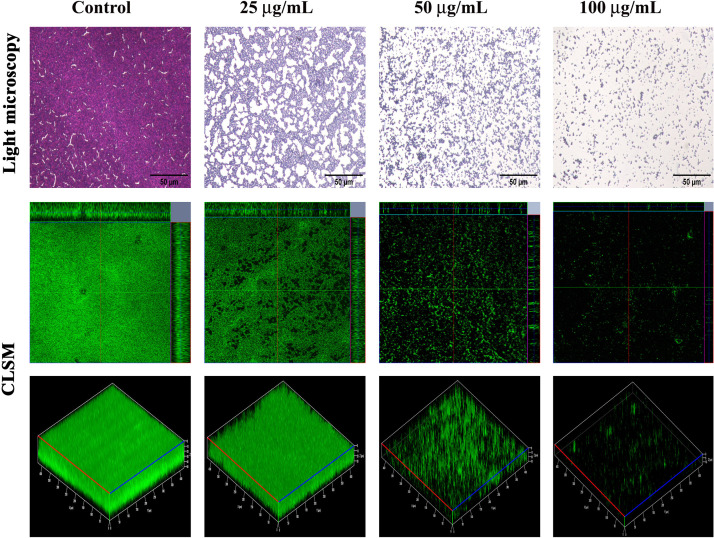
Light microscopic images (400×) and ortho- and three-dimensional confocal laser scanning microscopy images (200×) depicting the dose-dependent antibiofilm potential of thymol against methicillin-resistant *Staphylococcus aureus*. Scale bar = 50 μm in the light micrographs.

**FIGURE 3:**
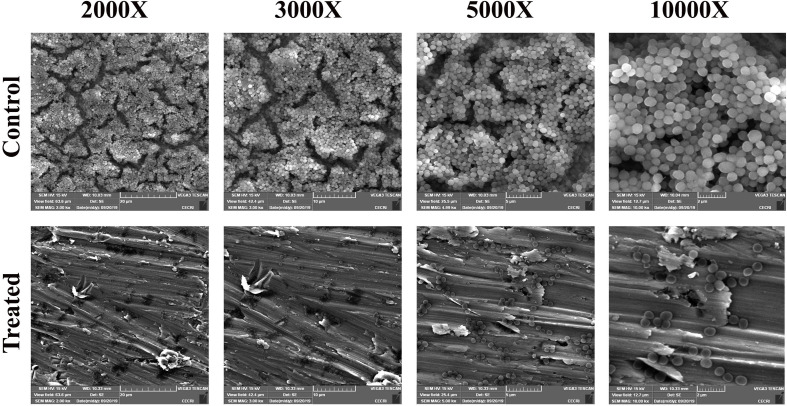
SEM images showing the reduction in adherence of methicillin-resistant *Staphylococcus aureus* on plasma-coated titanium surface upon thymol treatment (100 μg/ml) at different magnifications.

After confirming the non-antibacterial nature of thymol, the antibiofilm efficacy of thymol was further assessed by microscopic analysis. Light micrographs showed the gradual reduction in surface coverage with increasing concentrations of thymol. Furthermore, CLSM micrographs confirmed the ability of thymol to inhibit the surface adherence of MRSA, and notably, the thickness of the biofilm was also reduced upon increasing concentrations of thymol treatment (Figure 2). The human-plasma-coated titanium slides were subjected to MRSA biofilm formation in the absence and the presence of thymol and observed under SEM. The control titanium surface was observed to be completely covered by three-dimensional MRSA biofilm, whereas a monolayer of dispersed MRSA cells was observed in the thymol-treated titanium surface (Figure 3).

### *sarA*-Dependent Antibiofilm Activity of Thymol

**FIGURE 4:**
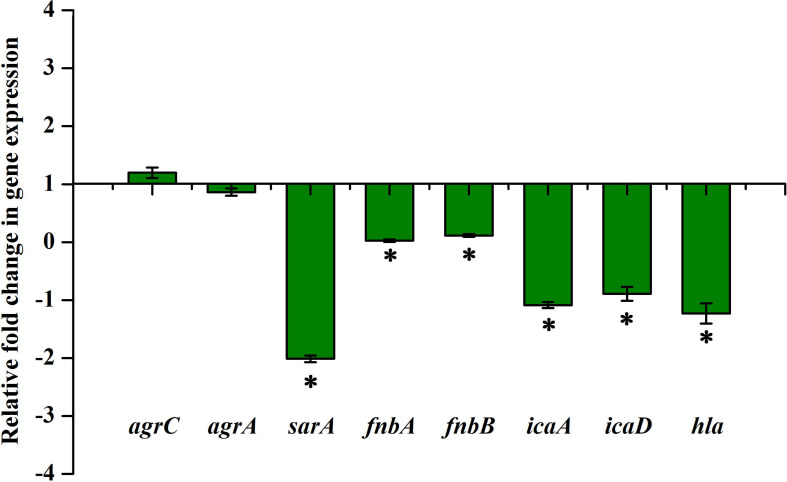
qPCR analysis of the expression of biofilm and virulence-associated genes after 24 h of thymol treatment. The error bars indicate standard deviations. The asterisks represent statistical significance (*p* < 0.05).

**FIGURE 5:**
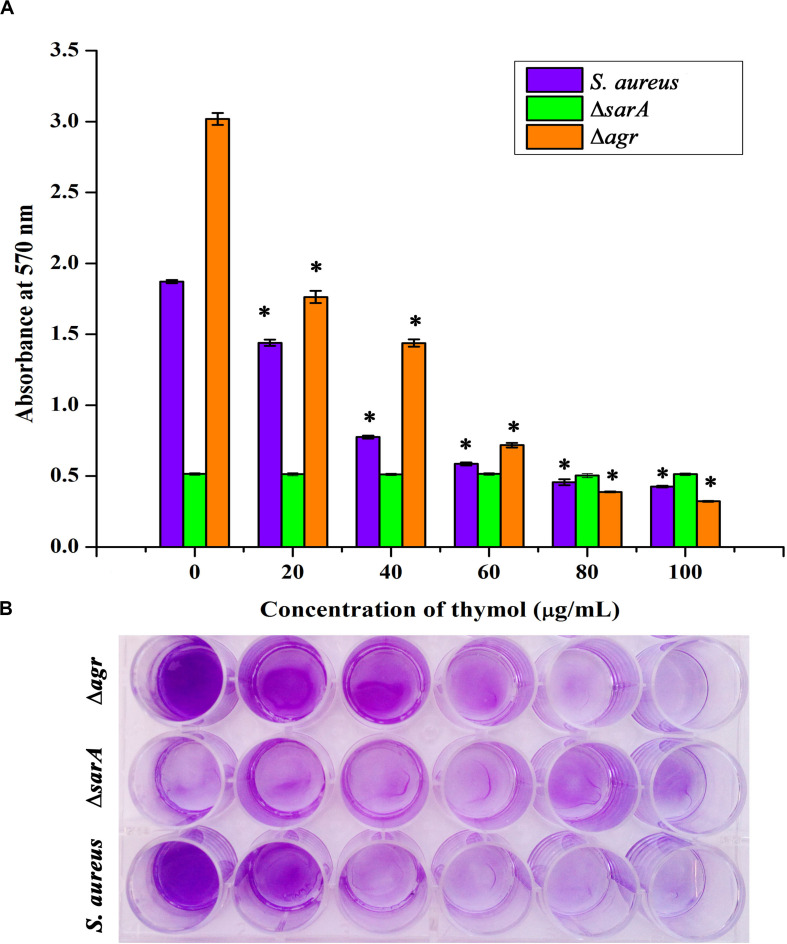
**(A)** Antibiofilm efficacy of thymol on wild-type *Staphylococcus aureus* and Δ*agr* (affected) and on Δ*sarA* (not affected) as depicted by crystal violet quantification of biofilm. **(B)** The 24-well microtiter plate assay depicting the effect of thymol on the biofilm formation of wild-type *S. aureus*, Δ*agr*, and Δ*sarA*. The error bars indicate standard deviations. The asterisks represent statistical significance (*p* < 0.05).

The expression of important regulatory genes involved in biofilm formation in the presence of thymol was examined by qPCR analysis. The expression of *sarA* was found to be decreased by twofold, whereas the expression of *agrA* and *agrC* was found to be unaltered by thymol treatment (Figure 4). Furthermore, the decreased expression of *sarA*-regulated virulence genes such as *fnbA*, *fnbB*, *icaA*, *icaD*, and *hla* was observed. The expression of *fnbA* and *fnbB* was slightly reduced by 0.02- and 0.11-fold, respectively, and nearly onefold down-regulation was observed in the expression of other virulence genes [*icaA* (1.08), *icaD* (0.89), and *hla* (1.23), respectively]. In order to identify the molecular-level target, the effect of thymol on biofilm formation of *S. aureus* wild type and Δ*sarA* and Δ*agr* strains was examined by crystal violet quantification assay as mentioned earlier. Biofilm quantification using OD_570 nm_ revealed that thymol was able to inhibit the biofilm formation of *S. aureus* wild type and Δ*agr* strains at 100 μg/ml. However, the biofilm formation by Δ*sarA* was found to be unaffected by thymol treatment (Figures 5A,B).

### Thymol Hampers *sarA*-Controlled Virulence in MRSA

**FIGURE 6:**
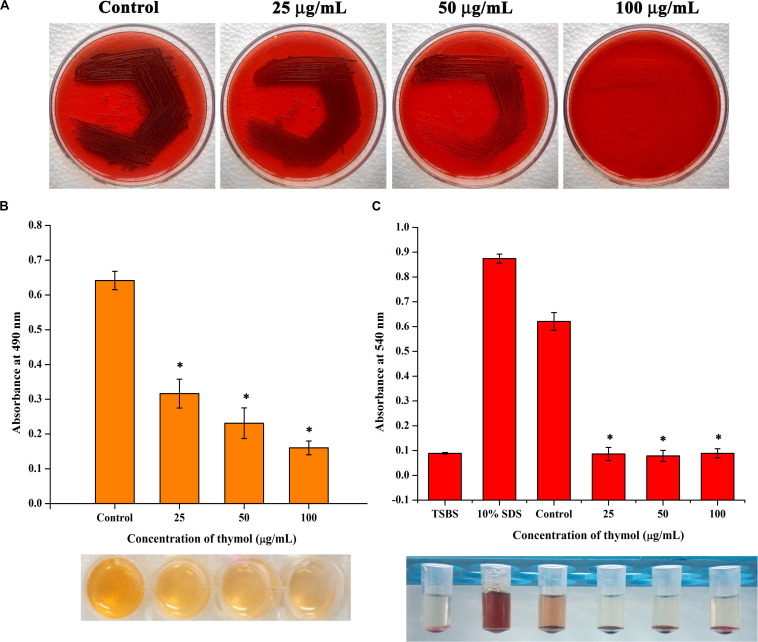
**(A)** Congo red assay representing the reduction in polysaccharide intracellular adhesion synthesis upon thymol treatment. **(B)** Thymol inhibits extracellular polysaccharide synthesis as revealed by phenol sulfuric acid quantification. **(C)** Thymol greatly inhibits the production of hemolysin as exhibited by erythrocyte lysis assay. The bottom image shows the corresponding assay tubes. The error bars indicate standard deviations. The asterisks represent statistical significance (*p* < 0.05).

**FIGURE 7:**
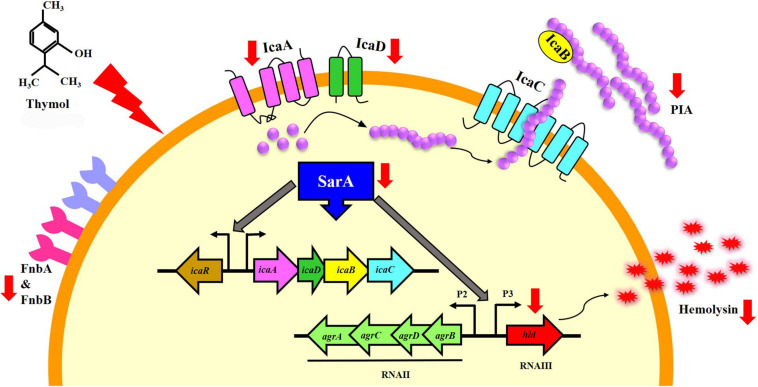
Schematic representation of the molecular mechanism of thymol mediated by *sarA*. The red arrows indicate the down-regulation of *sarA*-regulated virulence genes by thymol.

In the congo red agar (CRA) assay, the MRSA colonies appeared black in the absence of thymol; thymol treatment gradually inhibited the black coloration, and at 100 μg/ml, the MRSA cells appeared white, with complete inhibition of polysaccharide intracellular adhesion (PIA) (Figure 6A). Extracellular polysaccharide (EPS) present in MRSA biofilm was quantified by phenol-sulfuric acid method of carbohydrate quantification, and a significant reduction in EPS was observed at OD_490 nm_ in the thymol-treated samples when compared to the control sample (Figure 6B). In the erythrocyte lysis assay, the production of hemolysin was assessed by mixing CFCS of the control and the thymol-treated MRSA with human erythrocytes. The thymol treatment resulted in the complete inhibition of erythrocyte lysis when compared to the red-colored erythrocyte lysis in the control sample (Figure 6C). On the whole, thymol significantly reduced the virulence factors under the control of *sarA* which is clearly depicted in the schematic representation (Figure 7).

### Thymol Improves the Antibacterial Activity of Rifampicin Against Planktonic Cells, Mature Biofilm, and Persister Formation of MRSA

#### Antibacterial Activity of Rifampicin on Planktonic Cells

**FIGURE 8:**
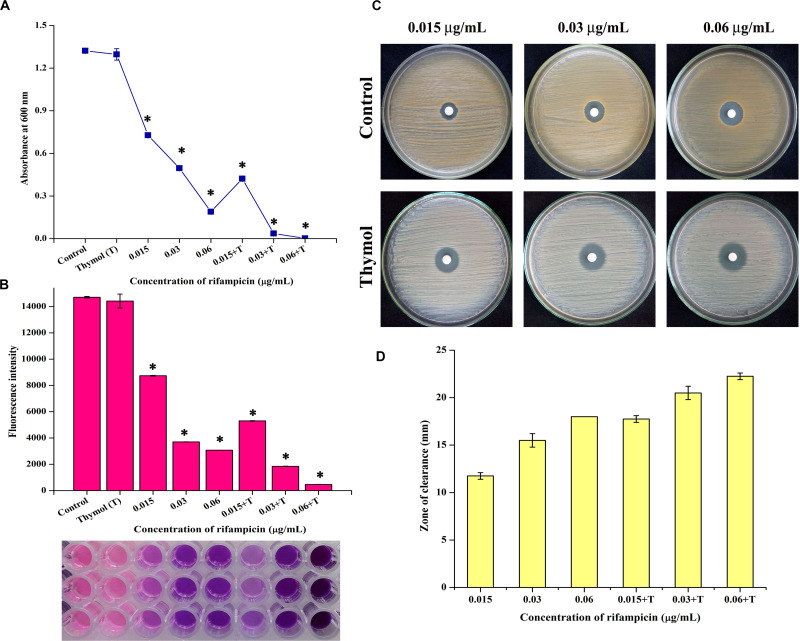
Thymol enhances the antibacterial activity of rifampicin as shown by growth optical density measurement **(A)**, Alamar blue assay **(B)**, disc diffusion assay **(C)**, and its zone of clearance **(D)**. The error bars indicate standard deviations. The asterisks represent statistical significance (*p* < 0.05).

The minimum inhibitory concentration (MIC) of thymol and rifampicin was determined as 160 and 0.06 μg/ml, respectively (Supplementary Figures S3, S4), as maximum growth was inhibited at the minimum concentrations. Hence, for the combinatorial assay, the BIC of thymol (100 μg/ml) was selected as it does not affect the growth of MRSA. The antibacterial activity of rifampicin at MIC (0.06 μg/ml) and at sub-MIC (0.015 and 0.03 μg/ml) concentrations in the presence of thymol was examined by growth OD measurement (Figure 8A) and Alamar blue-based viability assay (Figure 8B). The results demonstrated the enhanced reduction in growth as well as viability upon combinatorial treatment with rifampicin and thymol than treatment with rifampicin alone. In addition, images of disc diffusion assay (Figure 8C) clearly displayed the increased zone of clearance at all the tested concentrations of rifampicin in the presence of thymol. The zone of clearance (mm) of each plate was measured and presented as bar graph (Figure 8D). Interestingly, the MRSA cells appeared white in color in the TSA plates containing thymol because of staphyloxanthin inhibition, in contrast to the yellow-colored cells on the control TSA plates.

#### Time Killing Kinetics and Persister Formation of Rifampicin

**FIGURE 9:**
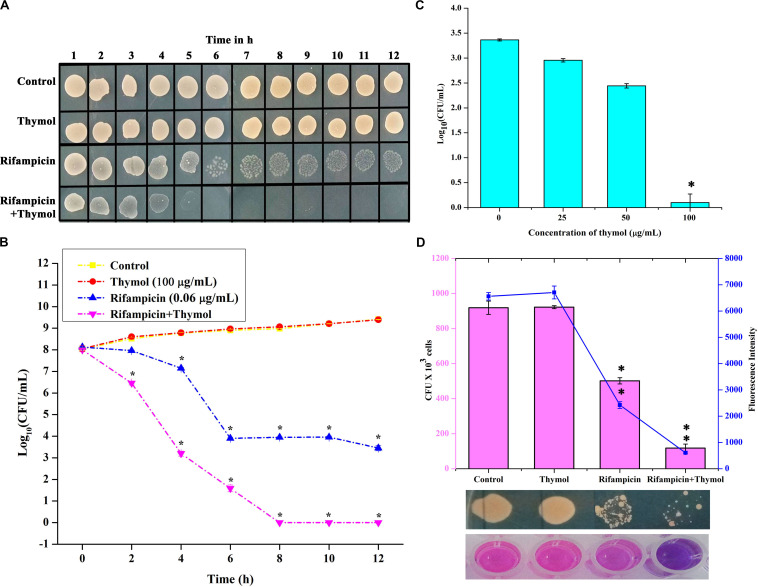
Thymol potentiates the killing efficacy of rifampicin as shown by time killing kinetics studied by spot assay **(A)** and colony-forming unit (CFU) counting **(B)** and reduces persister formation **(C)**. Thymol improves the biofilm eradication ability of rifampicin as shown by CFU analysis and Alamar blue assay **(D)**. The error bars indicate standard deviations. The asterisks represent statistical significance (*p* < 0.05).

The killing efficacy of rifampicin in the absence and the presence of thymol on overnight-grown MRSA culture was measured for 12 h by spot assay and CFU counting (Figures 9A,B). From the results, it is clear that although rifampicin reduced the number of surviving cells for first 6 h, a certain subpopulation of cells remained viable for 12 h. In the case of the combinatorial treatment with rifampicin and thymol, the entire population was killed within 6 h. Furthermore, a concentration-dependent reduction of persister formation was observed upon combinatorial treatment when compared to the persisters formed upon treatment with ×100 concentration of rifampicin alone (Figure 9C). The 100-μg/ml concentration of thymol especially completely inhibited the persister formation by rifampicin.

#### Antibacterial Activity of Rifampicin on Mature Biofilm

The effect of thymol on the antibacterial activity of rifampicin against the mature biofilm of MRSA was assessed. The viability of 24-h mature biofilm cells after a 12-h treatment with rifampicin and thymol alone and in combination was examined by CFU and Alamar blue assay (Figure 9D). The results of both assays unveiled that thymol treatment enhanced the antibacterial activity of rifampicin on preformed MRSA biofilm.

## Discussion

Thymol drastically inhibited the *in vitro* biofilm formation of *S. aureus* strains (MRSA ATCC strain and clinical isolates) at 100 μg/ml without affecting the growth and the metabolic viability. This is more advantageous in excluding the possibility of drug resistance as thymol did not apply any selection pressure on MRSA. Surface adherence in the form of biofilm provides a protective stay to MRSA from adverse conditions. Light and CLSM microscopic analyses further confirmed the ability of thymol to interfere with surface adherence of MRSA on glass and stainless steel slides. MRSA was reported to be the predominant human pathogen involved in infections associated with various implantable medical devices (Pinto et al., 2019). The adhesion of host matrix proteins on implant surfaces serves as an initiative factor for biofilm formation. MRSA produces specialized adhesive proteins that are named as microbial surface components recognizing adhesive matrix molecules, which could interact with host proteins (Foster and Höök, 1998). Thus, titanium surface coated with human plasma was taken for biofilm assays to mimic this natural phenomenon. As evidenced by SEM micrographs, a multilayered biofilm, with three-dimensional microcolonies, was observed in the control surface coated with plasma, whereas thymol treatment efficiently inhibited the adherence of MRSA even on plasma-coated titanium surface. The antibiofilm efficacy observed on various surfaces such as polystyrene, glass, stainless steel, and titanium irrespective of the nature of the surface makes thymol a better antibiofilm therapeutic candidate.

In order to find out the molecular mechanism of thymol at the transcriptional level, the expression of important biofilm regulatory genes such as *sarA*, *agrA*, and *agrC* was examined in the presence of thymol. SarA is basically a DNA binding protein which regulates the expression of genes involved in pathogenesis and stands as the global regulator of virulence in *S. aureus* (Bayer et al., 1996). On the other hand, *agr* is a two-component regulatory system which regulates toxin production and adhesion according to the quorum of bacteria present in a particular environment. An active *agr* system turns on the toxin gene expression and suppresses the adhesion gene expression (Boles and Horswill, 2008). Previous research on DNA binding sites of SarA revealed that SarA binds to the intergenic region between P2 and P3 promoters of the *agr* system and activates the expression of hemolysin *via* the RNAIII transcript. Apart from this, SarA binds to the upstream promoter regions of several target genes encoding fibronectin binding proteins (FnbA & FnbB), protein A (Spa), enterotoxin C, and PIA synthesis proteins (Chan and Foster, 1998; Chien et al., 1999). It is well known that *sarA* regulates the virulence of MRSA in both *agr*-dependent and *agr*-independent manner and serves as the therapeutic target to attenuate the virulence. Notably, previous studies have shown the *sarA* based repression of biofilm formation by various drugs in MRSA (Arya and Princy, 2013; Arya et al., 2015; Balamurugan et al., 2015, 2017). In the present study, thymol reduced the expression of *sarA* but did not alter the expression of *agrA* and *agrC*. This result leads to the hypothesis that the antibiofilm activity of thymol could be *sarA*-mediated. To verify this hypothesis, the antibiofilm activity of thymol was assessed on Δ*sarA* and Δ*agr* strains. It was already reported that *sarA* mutation reduces the ability of *S. aureus* to form a biofilm, whereas *agr* mutation induces biofilm formation. In the present study also, Δ*sarA* formed less biofilm than that of the wild-type strain. As predicted, thymol was found to be ineffective on Δ*sarA*, and interestingly, thymol effectively inhibited the biofilm formation by Δ*agr*. The inefficacy of thymol on Δ*sarA* validated the *sarA*-dependent antibiofilm activity of thymol.

To further prove this mechanism, the effect of thymol on the transcription of *sarA*-controlled virulence genes was investigated. The result revealed the down-regulation of *sarA*-controlled virulence genes such as *icaA*, *icaD*, *fnbA*, *fnbB*, and *hla*. PIA produced by *ica* operon plays a vital role in MRSA biofilm formation. It is well reported that the expression of the *ica* locus is positively regulated by *sarA* in *S. aureus* (Cerca et al., 2008). The PIA polymer is synthesized in the cytoplasm and transported outside the cell, which then mediates the intracellular adhesion and thereby provides the structural stability to biofilm (O’Gara, 2007). The reduction in the expression of *icaA* and *icaD* was further confirmed by CRA assay as it is an important method to identify the PIA production and *ica*-positive *S. aureus* by means of black coloration. The results of the CRA assay revealed that, as a downstream impact of *sarA* inhibition by thymol, the treated cells appeared white in color due to the complete inhibition of PIA synthesis. Phenol-sulfuric acid quantification of EPS also evidenced that thymol inhibited the EPS present in the biofilm matrix. Hemolysin is an important pore-forming virulence factor produced by *S. aureus* and plays a crucial role in invasive staphylococcal infections. The synthesis of hemolysin was affected by *sarA* inhibitors in previous studies (Arya et al., 2015; Balamurugan et al., 2017). In the present study also, the inhibition of *sarA* expression by thymol ultimately reduced the production of hemolysin, as exhibited by the erythrocyte lysis assay. The *in vitro* assays confirmed that the reduction in *sarA* expression could be the central mechanism involved in the antibiofilm activity of thymol, and a schematic representation of the molecular mechanism of thymol is depicted in Figure 7. Rifampicin, also known as rifampin, is the broad-spectrum antibiotic used to treat several microbial infections and commonly used to treat skin infections, prosthetic joint infections, and biofilm-associated infections caused by *S. aureus* (Lebeaux et al., 2014). Mostly, rifampicin is included in the combinatorial treatment strategy because of its ability to easily penetrate cells, and the development of resistance against rifampicin can also be minimized (Chang et al., 2020). Thus, the present study evaluated the efficacy of thymol to enhance the antibacterial efficacy of rifampicin against MRSA and found a great reduction in the growth and the viability of MRSA upon combinatorial treatment. The increased zone of clearance in the disc diffusion assay and the enhanced killing efficacy with less time span (6 h) confirmed the potential of the combinatorial treatment. Persisters are antibiotic-tolerant bacterial cells but different from antibiotic-resistant mutants since the antibiotic tolerance of persisters is not inheritable and reversible. Persisters play a critical role in the recurrence of a bacterial infection after the course of an antibiotic treatment, and this condition is clinically challenging (Lechner et al., 2012). Interestingly, thymol was found to be effective in reducing persister formation upon rifampicin treatment, and it would be of great advantage in the clinical settings. The eradication of preformed biofilm on medical devices is another hard task which could not be done with the available antibiotics, and possibly the combinatorial treatment of antibiotics and antibiofilm agents could solve this critical issue (Roy et al., 2013). Thus, the effect of combinatorial treatment on mature biofilm was evaluated in the present study. The number of viable bacteria in the mature biofilm after treatment with the rifampicin and thymol combination was greatly reduced than the rifampicin-alone-treated sample as revealed by CFU analysis and Alamar blue viability assay. Thus, all these results strongly suggest that thymol has the ability to boost the antibacterial activity of rifampicin in the form of combinatorial treatment against planktonic, biofilm, and persister cells of MRSA, and this property of thymol is highly appreciable in terms of clinical applications.

## Conclusion

The present study unveiled the *sarA*-dependent antibiofilm activity and inhibition of other virulence factors such as PIA and hemolysin synthesis by thymol. The inhibition of MRSA adherence on various surfaces such as polystyrene, glass, stainless steel, and plasma-coated titanium advocates the potential of thymol as a surface-independent antibiofilm candidate in clinical context. The possibility of resistance development is also meager as thymol exerts non-antibacterial antibiofilm activity at 100 μg/ml. Thymol potentiated the antibacterial activity of rifampicin on planktonic as well as biofilm cells and reduced the persister formation. The ability of thymol to enhance the antibacterial and the biofilm eradication efficiency of rifampicin makes it a promising therapeutic candidate for combinatorial treatment strategy.

## Data Availability Statement

All datasets generated for this study are included in the article/Supplementary Material.

## Ethics Statement

The studies involving human participants were reviewed and approved by Institutional Ethical Committee, Alagappa University, Karaikudi (IEC Ref No: IEC/AU/2016/1/4). The patients/participants provided their written informed consent to participate in this study.

## Author Contributions

AV and SK designed the study. AV, AS, and UY performed the experiments. AV analyzed the data, prepared the figures and tables, and wrote the manuscript. AS performed the statistical analysis. SK and CA revised the manuscript. All authors have read and approved the final version of the manuscript.

## Conflict of Interest

The authors declare that the research was conducted in the absence of any commercial or financial relationships that could be construed as a potential conflict of interest.

## Abbreviations

*agr*: accessory gene regulator
BIC: biofilm inhibitory concentration
CFCS: cell-free culture supernatant
CFU: colony forming unit
CLSM: confocal laser scanning microscope
CRA: congo red agar
EPS: extracellular polysaccharide
MRSA: methicillin-resistant *Staphylococcus aureus;* MTP microtiter plate
PIA: polysaccharide intracellular adhesion
*sarA*: staphylococcal accessory regulator A
SEM: scanning electron microscopy
TSBS: tryptone soya broth supplemented with 1% sucrose
WHO: World Health Organization.

## References

[B1] Abraham K. P. Srieenivas J. Venkateswarulu T. C. Indira M. Babu D. J. Diwakar T. (2012). Investigation of the potential anti biofilm activities of plant extracts. *Int. J. Pharm. Pharm. Sci.* 4 282–285.

[B2] Arya R. Princy S. A. (2013). Computational approach to design small molecule inhibitors and identify *SarA*as a potential therapeutic candidate. *Med. Chem. Res.* 22 1856–1865. 10.1007/s00044-012-0185-9

[B3] Arya R. Ravikumar R. Santhosh R. S. Princy S. A. (2015). *SarA*based novel therapeutic candidate against *Staphylococcus aureus*associated with vascular graft infections. *Front. Microbiol.* 6:416. 10.3389/fmicb.2015.00416 26074884 PMC4447123

[B4] Azizi Z. Ebrahimi S. Saadatfar E. Kamalinejad M. Majlessi N. (2012). Cognitive-enhancing activity of thymol and carvacrol in two rat models of dementia. *Behav. Pharmacol.* 23 241–249. 10.1097/FBP.0b013e3283534301 22470103

[B5] Balamurugan P. Hema M. Kaur G. Sridharan V. Prabu P. C. Sumana M. N. (2015). Development of a biofilm inhibitor molecule against multidrug resistant *Staphylococcus aureus*associated with gestational urinary tract infections. *Front. Microbiol.* 6:832. 10.3389/fmicb.2015.00832 26322037 PMC4531255

[B6] Balamurugan P. Praveen Krishna V. Bharath D. Lavanya R. Vairaprakash P. Adline Princy S. (2017). *Staphylococcus aureus*Quorum regulator *sarA*targeted compound, 2-[(Methylamino) methyl] phenol inhibits biofilm and down-regulates virulence genes. *Front. Microbiol.* 8:1290. 10.3389/fmicb.2017.01290 28744275 PMC5504099

[B7] Bayer M. G. Heinrichs J. H. Cheung A. L. (1996). The molecular architecture of the sar locus in *Staphylococcus aureus*. *J. Bacteriol.* 178 4563–4570. 10.1128/jb.178.15.4563-4570.1996 8755885 PMC178224

[B8] Bernheimer A. W. (1988). Assay of hemolytic toxins. *Methods Enzymol.* 165 213–217. 10.1016/s0076-6879(88)65033-6 2906728

[B9] Boles B. R. Horswill A. R. (2008). Agr-mediated dispersal of *Staphylococcus aureus*biofilms. *PLoS Pathog.* 4:e1000052. 10.1371/journal.ppat.1000052 18437240 PMC2329812

[B10] Braga P. C. (2005). Thymol: antibacterial, antifungal and antioxidant activities. *G. Ital. Ostet. Ginecol.* 27 267–272.

[B11] Buommino E. Scognamiglio M. Donnarumma G. Fiorentino A. D’Abrosca B. (2014). Recent advances in natural product-based anti-biofilm approaches to control infections. *Mini Rev. Med. Chem.* 14 1169–1182. 10.2174/1389557515666150101095853 25553429

[B12] Cerca N. Brooks J. L. Jefferson K. K. (2008). Regulation of the intercellular adhesin locus regulator (*icaR*) by SarA, σB, and IcaR in *Staphylococcus aureus*. *J. Bacteriol.* 190 6530–6533. 10.1128/JB.00482-08 18658265 PMC2565999

[B13] Chan P. F. Foster S. J. (1998). Role of SarA in virulence determinant production and environmental signal transduction in *Staphylococcus aureus*. *J. Bacteriol.* 180 6232–6241. 10.1128/.180.23.6232-6241.1998 9829932 PMC107708

[B14] Chang A. T. Cosimi R. A. Bochan M. R. (2020). Treatment of Staphylococcal Device infections: synergistic daptomycin with ceftaroline versus rifampin-adjunct therapy. *Open Forum Infect. Dis.* 7:ofaa072. 10.1093/ofid/ofaa072 32195290 PMC7075485

[B15] Chien Y. Manna A. C. Projan S. J. Cheung A. L. (1999). SarA, a global regulator of virulence determinants in *Staphylococcus aureus*, binds to a conserved motif essential for sar-dependent gene regulation. *J. Biol. Chem.* 274 37169–37176. 10.1074/jbc.274.52.37169 10601279

[B16] Dubois M. Gilles K. Hamilton J. K. Rebers P. A. Smith F. (1951). A colorimetric method for the determination of sugars. *Nature* 168:167. 10.1038/168167a0 14875032

[B17] Foster T. J. Höök M. (1998). Surface protein adhesins of *Staphylococcus aureus*. *Trends Microbiol.* 6 484–488. 10.1016/s0966-842x(98)01400-0 10036727

[B18] García-Salinas S. Elizondo-Castillo H. Arruebo M. Mendoza G. Irusta S. (2018). Evaluation of the antimicrobial activity and cytotoxicity of different components of natural origin present in essential oils. *Molecules* 23:1399. 10.3390/molecules23061399 29890713 PMC6100501

[B19] Gowrishankar S. Duncun Mosioma N. Karutha Pandian S. (2012). Coral-associated bacteria as a promising antibiofilm agent against methicillin-resistant and-susceptible *Staphylococcus aureus*biofilms. *Evid. Based Complementary Altern.* 2012:862374. 10.1155/2012/862374 22988476 PMC3439993

[B20] Hall C. W. Mah T.-F. (2017). Molecular mechanisms of biofilm-based antibiotic resistance and tolerance in pathogenic bacteria. *FEMS Microbiol. Rev.* 41 276–301. 10.1093/femsre/fux010 28369412

[B21] Jenul C. Horswill A. R. (2018). Regulation of *Staphylococcus aureus*virulence. *Microbiol. Spectr.* 6:1. 10.1128/microbiolspec.GPP3-0031-2018 30953424 PMC6452892

[B22] Knobloch J. K.-M. Horstkotte M. A. Rohde H. Mack D. (2002). Evaluation of different detection methods of biofilm formation in *Staphylococcus aureus*. *Med. Microbiol. Immunol.* 191 101–106. 10.1007/s00430-002-0124-3 12410349

[B23] Kumar S. Chandra N. Singh L. Hashmi M. Z. Varma A. (2019). *Biofilms in Human Diseases: Treatment and Control.* Cham: Springer.

[B24] Lebeaux D. Ghigo J.-M. Beloin C. (2014). Biofilm-related infections: bridging the gap between clinical management and fundamental aspects of recalcitrance toward antibiotics. *Microbiol. Mol. Biol. Rev.* 78 510–543. 10.1128/MMBR.00013-14 25184564 PMC4187679

[B25] Lechner S. Lewis K. Bertram R. (2012). *Staphylococcus aureus*persisters tolerant to bactericidal antibiotics. *J. Mol. Microbiol. Biotechnol.* 22 235–244. 10.1159/000342449 22986269 PMC3518770

[B26] Lee J.-H. Kim Y.-G. Gwon G. Wood T. K. Lee J. (2016). Halogenated indoles eradicate bacterial persister cells and biofilms. *AMB Express* 6:123. 10.1186/s13568-016-0297-6 27921270 PMC5138170

[B27] Livak K. J. Schmittgen T. D. (2001). Analysis of relative gene expression data using real-time quantitative PCR and the 2(-Delta Delta C(T)) method. *Methods* 25 402–408. 10.1006/meth.2001.1262 11846609

[B28] Nostro A. Sudano Roccaro A. Bisignano G. Marino A. Cannatelli M. A. Pizzimenti F. C. (2007). Effects of oregano, carvacrol and thymol on *Staphylococcus aureus and Staphylococcus epidermidis*biofilms. *J. Med. Microbiol*. 56 519–523. 10.1099/jmm.0.46804-0 17374894

[B29] O’Gara J. P. (2007). ica and beyond: biofilm mechanisms and regulation in *Staphylococcus epidermidis* and *Staphylococcus aureus*. *FEMS Microbiol. Lett.* 270 179–188. 10.1111/j.1574-6968.2007.00688.x 17419768

[B30] Olsen I. (2015). Biofilm-specific antibiotic tolerance and resistance. *Eur. J. Clin. Microbiol. Infect. Dis. Off. Publ. Eur. Soc. Clin. Microbiol.* 34 877–886. 10.1007/s10096-015-2323-z 25630538

[B31] Palavecino E. L. (2014). Clinical, epidemiologic, and laboratory aspects of methicillin-resistant *Staphylococcus aureus*infections. *Methods Mol. Biol.* 1085 1–24. 10.1007/978-1-62703-664-1_1 31523762

[B32] Pinto R. M. Lopes-de-Campos D. Martins M. C. L. Van Dijck P. Nunes C. Reis S. (2019). Impact of nanosystems in *Staphylococcus aureus*biofilms treatment. *FEMS Microbiol. Rev.* 43 622–641. 10.1093/femsre/fuz021 31420962 PMC8038934

[B33] Poonacha N. Nair S. Desai S. Tuppad D. Hiremath D. Mohan T. (2017). Efficient killing of planktonic and biofilm-embedded coagulase-negative Staphylococci by bactericidal protein P128. *Antimicrob. Agents Chemother.* 61:e00457-17. 10.1128/AAC.00457-17 28559263 PMC5527639

[B34] Roy V. Meyer M. T. Smith J. A. I. Gamby S. Sintim H. O. Ghodssi R. (2013). AI-2 analogs and antibiotics: a synergistic approach to reduce bacterial biofilms. *Appl. Microbiol. Biotechnol.* 97 2627–2638. 10.1007/s00253-012-4404-6 23053069

[B35] Sarker S. D. Nahar L. Kumarasamy Y. (2007). Microtitre plate-based antibacterial assay incorporating resazurin as an indicator of cell growth, and its application in the *in vitro*antibacterial screening of phytochemicals. *Methods* 42 321–324. 10.1016/j.ymeth.2007.01.006 17560319 PMC1895922

[B36] Selvaraj A. Jayasree T. Valliammai A. Pandian S. K. (2019). Myrtenol attenuates MRSA biofilm and virulence by suppressing *sarA e*xpression dynamism. *Front. Microbiol.* 10:2027. 10.3389/fmicb.2019.02027 31551964 PMC6737500

[B37] Sethupathy S. Ananthi S. Selvaraj A. Shanmuganathan B. Vigneshwari L. Balamurugan K. (2017). Vanillic acid from *Actinidia deliciosa*impedes virulence in *Serratia marcescens*by affecting S-layer, flagellin and fatty acid biosynthesis proteins. *Sci. Rep.* 7:16328. 10.1038/s41598-017-16507-x 29180790 PMC5703977

[B38] Sethupathy S. Prasath K. G. Ananthi S. Mahalingam S. Balan S. Y. Pandian S. K. (2016). Proteomic analysis reveals modulation of iron homeostasis and oxidative stress response in *Pseudomonas aeruginosa*PAO1 by curcumin inhibiting quorum sensing regulated virulence factors and biofilm production. *J. Proteomics* 145 112–126. 10.1016/j.jprot.2016.04.019 27108548

[B39] Taylor T. A. Unakal C. G. (2020). *Staphylococcus Aureus.* Treasure Island, FL: StatPearls Publishing. 28722898

[B40] Tohidpour A. Sattari M. Omidbaigi R. Yadegar A. Nazemi J. (2010). Antibacterial effect of essential oils from two medicinal plants against Methicillin-resistant *Staphylococcus aureus*(MRSA). *Phytomedicine* 17 142–145. 10.1016/j.phymed.2009.05.007 19576738

[B41] Valliammai A. Sethupathy S. Priya A. Selvaraj A. Bhaskar J. P. Krishnan V. (2019). 5-Dodecanolide interferes with biofilm formation and reduces the virulence of Methicillin-resistant *Staphylococcus aureus*(MRSA) through up regulation of agr system. *Sci. Rep.* 9:13744. 10.1038/s41598-019-50207-y 31551455 PMC6760239

[B42] Vasudevan R. (2019). Agr/sarA: molecular switches of biofilm regulation in *Staphylococcus aureus*.J. *Microbiol. Exp.* 7 17–18. 10.15406/jmen.2019.07.00233

[B43] Walker J. Horswill A. (2012). A coverslip-based technique for evaluating *Staphylococcus aureus*biofilm formation on human plasma. *Front. Cell. Infect. Microbiol.* 2:39. 10.3389/fcimb.2012.00039 22919630 PMC3417647

