# Supplementary material for: sarA-Dependent Antibiofilm Activity of Thymol Enhances the Antibacterial Efficacy of Rifampicin Against Staphylococcus aureus
Source: Front Microbiol. 2020 Jul 31;11:1744. doi: 10.3389/fmicb.2020.01744 (PMC7410925; doi:10.3389/fmicb.2020.01744)

**Supplementary Material**

***sarA* dependent antibiofilm activity of thymol enhances the antibacterial efficacy of rifampicin against *Staphylococcus aureus***

Alaguvel Valliammai^1^, Anthonymuthu Selvaraj^1^, Udayakumar Yuvashree^1^, Chairmandurai Aravindraja^1,2^ and Shunmugiah Karutha Pandian^1^*

**Figure S1.** Effect of increasing concentrations of thymol on biofilm formation of clinical isolates of *S. aureus*. Error bars indicate standard deviations. Asterisks represent statistical significance (*p* < 0.05).


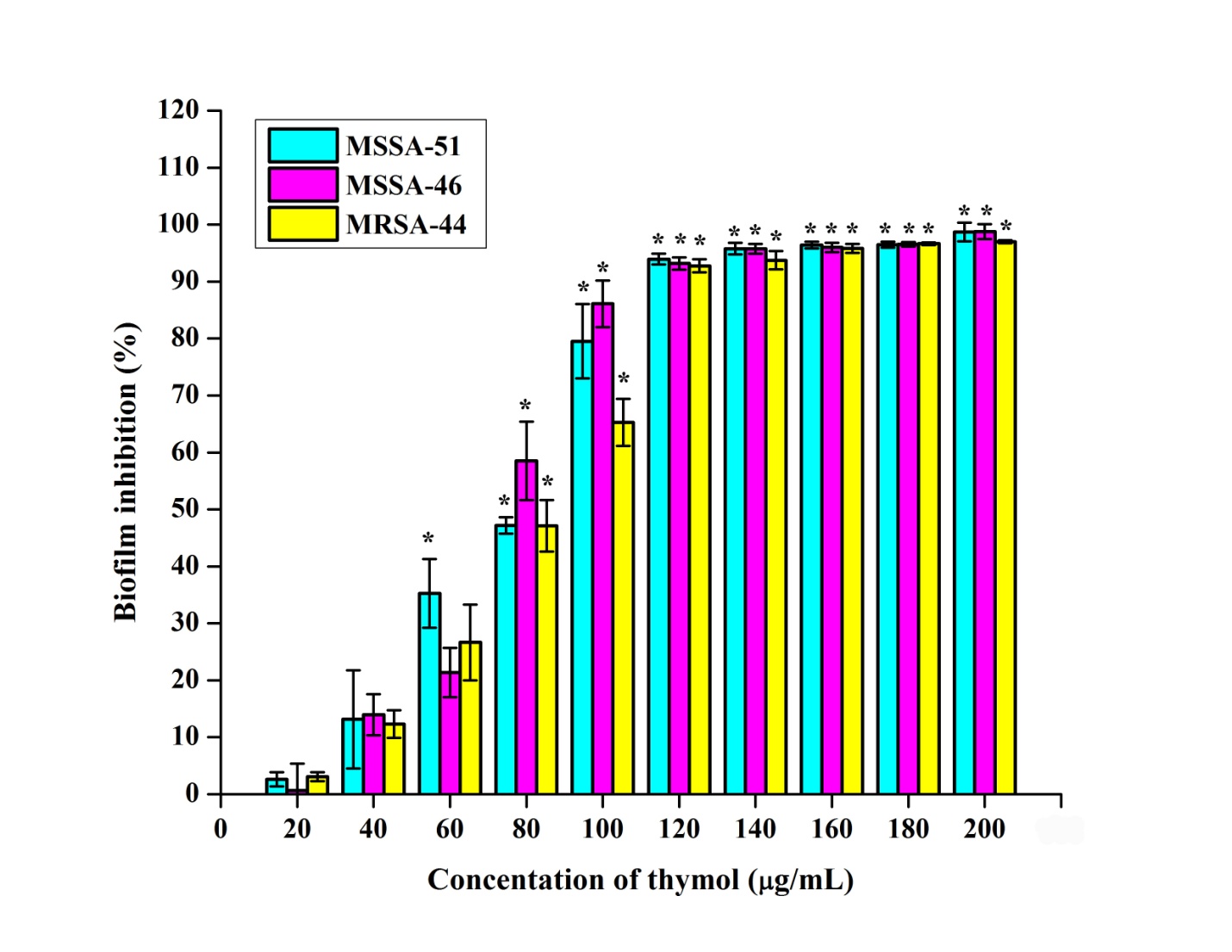


**Figure S2.** Effect of thymol on growth and metabolism of MRSA at BIC (100 µg/mL). (a) CFU analysis of control and thymol treated MRSA exhibiting non-antibacterial nature of thymol. (b) Alamar blue assay depicting the metabolic viability of control and thymol treated MRSA. Error bars indicate standard deviations.


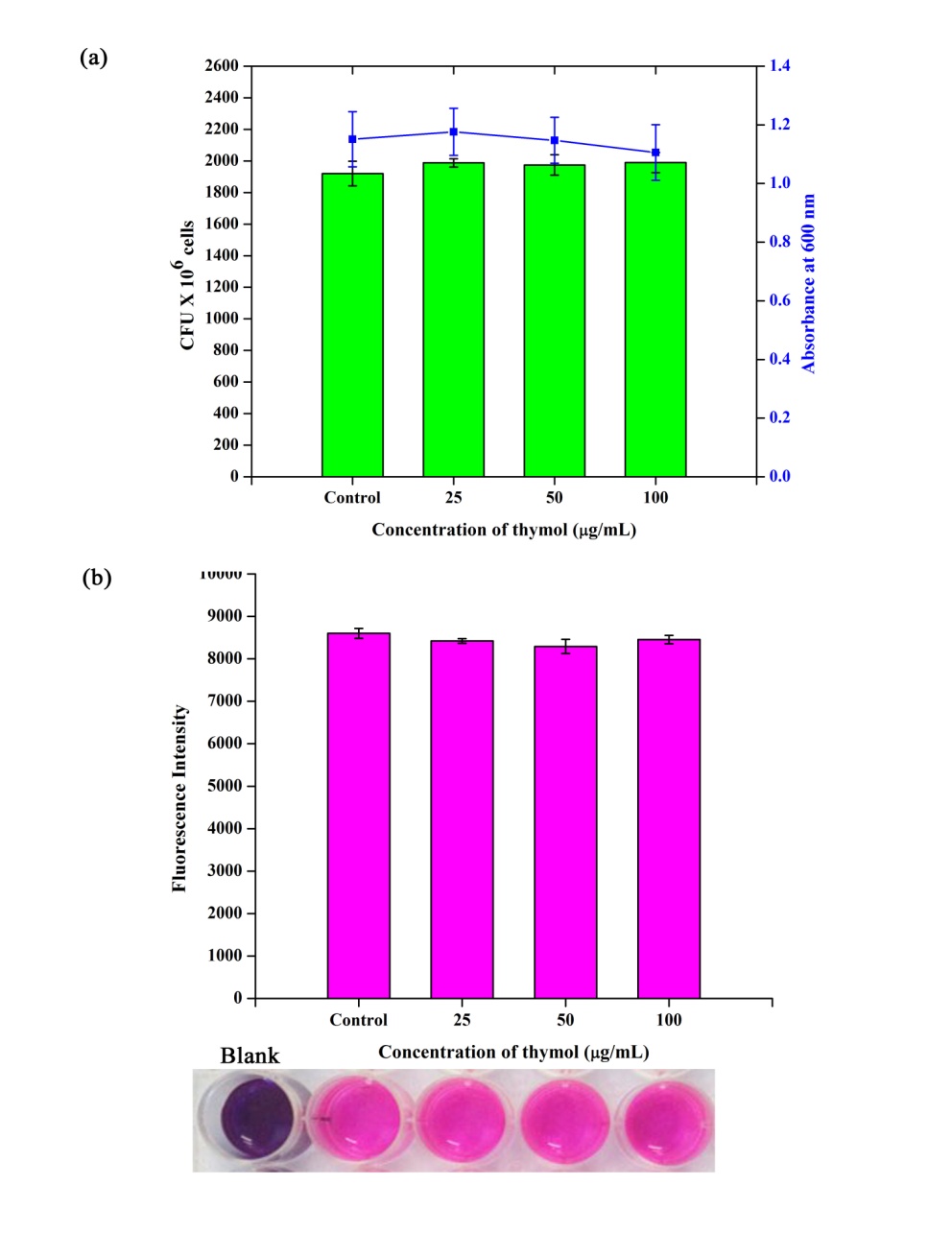


**Figure S3.** Determination of MIC of thymol against MRSA. Error bars indicate standard deviations. Asterisks represent statistical significance (*p* < 0.05).


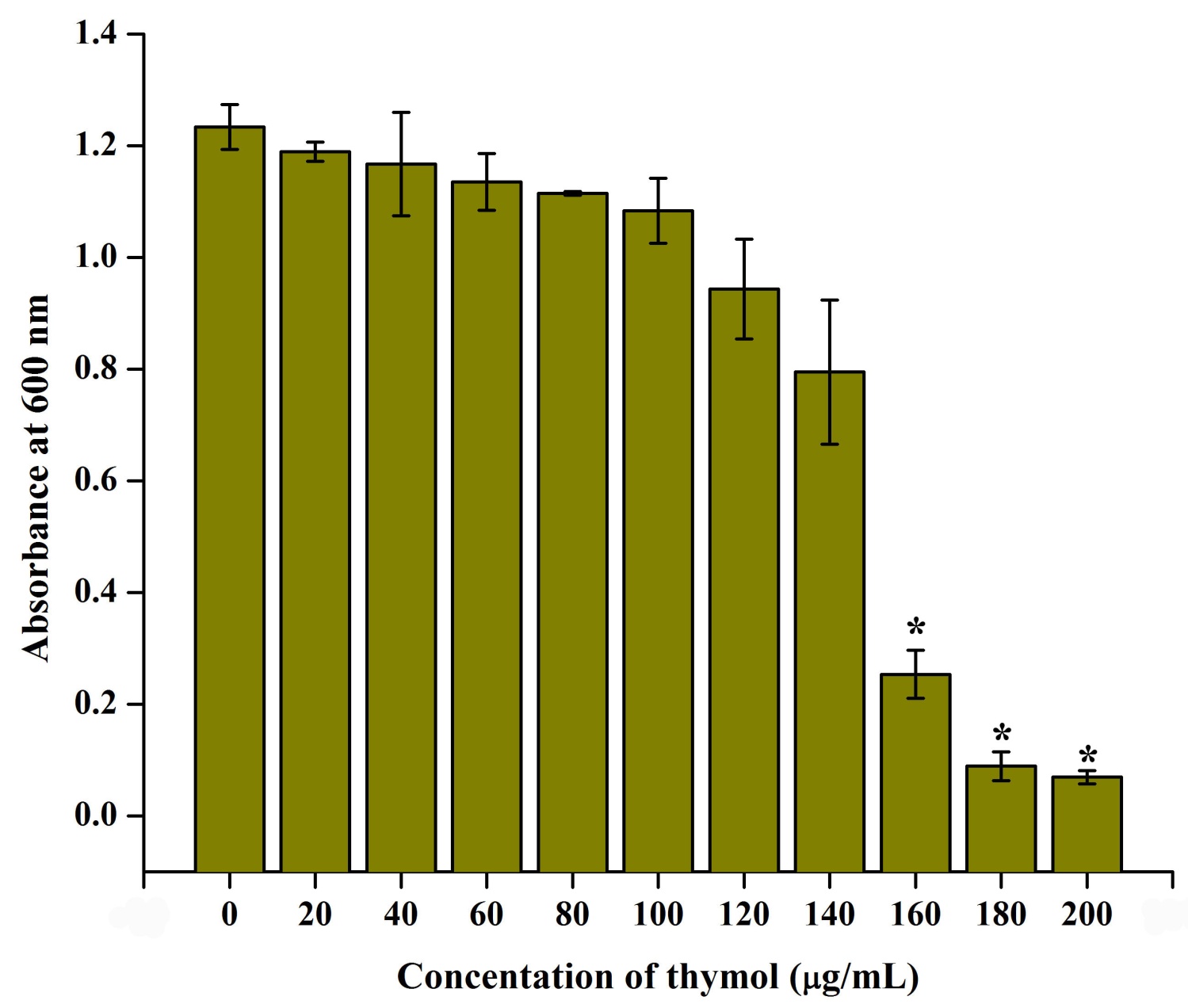


**Figure S4.** Determination of MIC of rifampicin against MRSA. Error bars indicate standard deviations. Asterisks represent statistical significance (*p* < 0.05).


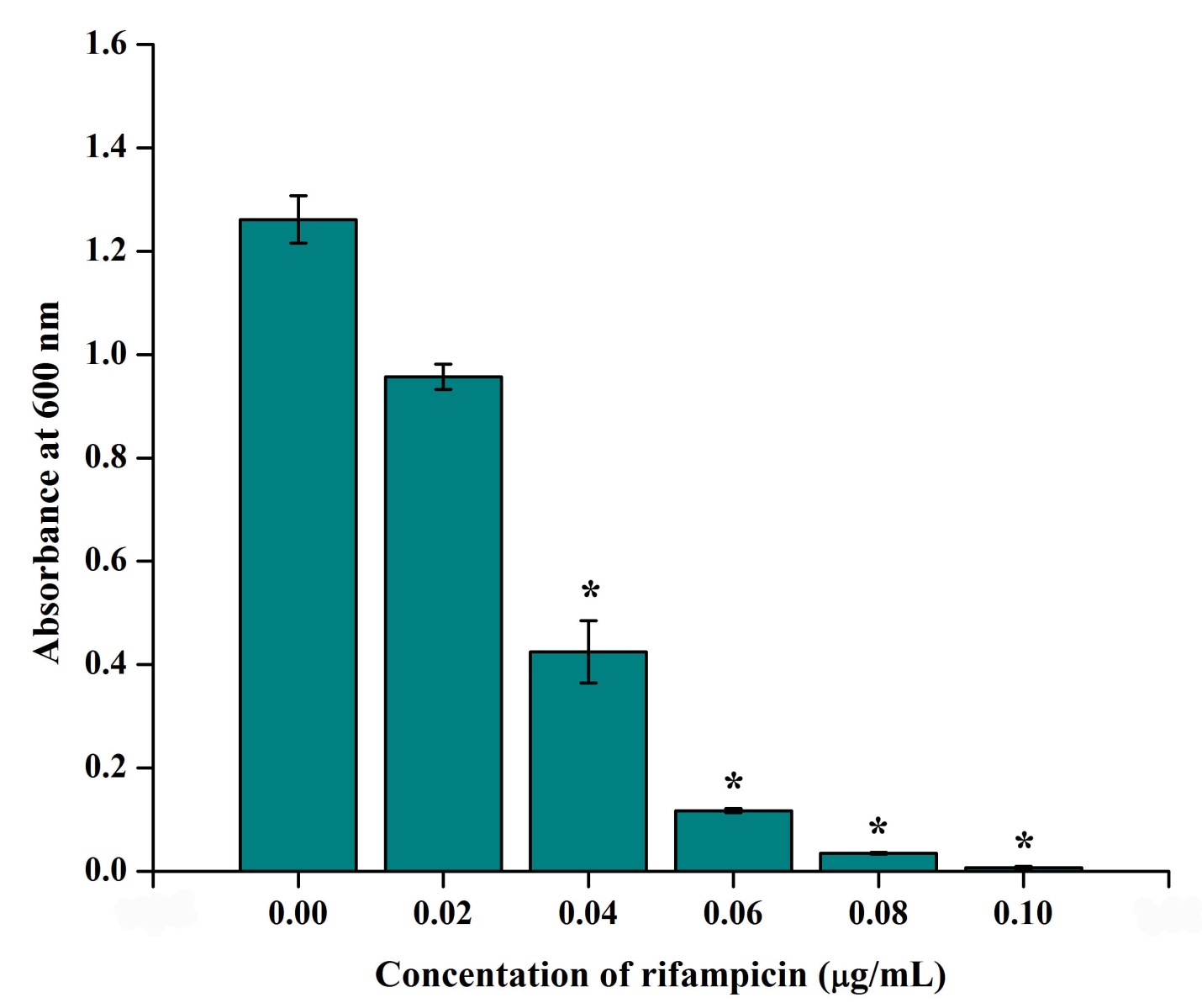

Supplement: Supplementary file 1 [file Data_Sheet_1.docx]
